# Therapy for Dupuytren’s Disease: Collagenase Therapy—A Long-Term Follow-Up Study

**DOI:** 10.3390/life14101275

**Published:** 2024-10-08

**Authors:** Nikolaus Wachtel, Francesca Romana Dingler, Tim Nürnberger, Felix Hubertus Vollbach, Nicholas Moellhoff, Riccardo Giunta, Wolfram Demmer

**Affiliations:** Department of Hand, Plastic and Aesthetic Surgery, LMU Klinikum, Ziemssenstraße 5, 80336 Munich, Germany

**Keywords:** Dupuytren’s disease, palmar fascial fibromatosis, Viking disease, contracture, collagenase, Xiapex

## Abstract

Background: Dupuytren’s disease (DD) is a systemic connective tissue disorder of the palm. It particularly affects men of Northern European or Caucasian origin over the age of 55. In addition to the classical surgical therapy via limited fasciectomy, Dupuytren’s contracture can also be treated minimally invasively. A relatively new treatment method is the use of collagenase injections (Xiapex) to reduce the contracture of the fingers. The data regarding the long-term success of this therapy are currently limited. Methods: In this monocentric retrospective study, we examined 35 patients who were treated with collagenase (Xiapex) for Dupuytren’s contracture in the long fingers. Following the manufacturer’s recommendations, the injection was administered intralesionally, and the cord was ruptured through the passive extension of the finger under local anesthesia with Mepivacain the following day. The clinical follow-up examination was conducted after an average of 5.7 years. The stages of Dupuytren’s disease were documented using the Tubiana classification. Additionally, parameters of finger extension ability, differentiated by metacarpophalangeal (MCP), and proximal interphalangeal (PIP) joints, as well as patient-specific risk parameters, were evaluated Results: The long-term results of collagenase therapy after an average of 5.7 years showed a significant improvement in the contracture of the affected fingers. In the MCP joints, the flexion contracture decreased from 42° to 17° (*p* ≤ 0.001), and in the PIP joints, it decreased from 56° to 33° (*p* ≤ 0.001). The primary recurrence rate was 11% for the MCP joints and 19% for the PIP joints, respectively. The analysis of risk factors showed a significant risk for worse long-term outcomes in patients with diabetes and those with nicotine abuse. Conclusions: Collagenase therapy for Dupuytren’s disease achieved significant long-term improvements in contracture in both MCP and PIP joints. In accordance with general risk factors for DD, patients with diabetes and those with nicotine abuse are at risk of worse long-term outcomes. Overall, it is a time-saving, low-risk, and straightforward technique for treating the disabling contracture component of this disease.

## 1. Introduction

Dupuytren’s disease (DD) is a benign, usually progressive, and painless fibrotic condition affecting the palmar fascia of the hand [1,2,3]. The condition predominantly affects men of Northern European or Caucasian descent, with a typical onset after the age of 55 [2,4]. Consequently, the condition is infrequently seen in Asian and African American populations, while in Western countries, the average prevalence is reported to be 12% among 55-year-olds and 29% among 75-year-olds [5,6,7,8]. Women are less frequently affected and generally display a milder course [4,6,9,10].

In its early stages, DD manifests as discrete nodules or dimples in the skin of the palm. In advanced stages, solid fibrous cords form along the finger rays, leading to flexion contractures known as “Dupuytren’s contracture” [4]. To describe the extent of the contracture caused by Dupuytren’s disease, Tubiana developed a classification (stage 0–4) in which the total contracture of all affected joints in one finger is measured in degrees of deviation from the neutral position [11,12,13,14].

The development of nodules and contractures in Dupuytren’s disease is caused by an increased production of collagen, especially type III. Both the overexpression of growth factors and increased tissue ischemia with subsequent myofibroblast proliferation are considered responsible for this [15,16].

In addition to an inherited genetic component, various risk factors are associated with an increased risk of developing Dupuytren’s disease, such as male gender, epilepsy, diabetes mellitus, alcohol abuse and liver diseases, nicotine abuse, vibration exposure and heavy manual labor, previous hand trauma or infection, low body mass index (BMI), hypercholesterolemia, and blue or green eye color [4,17]. However, these are still controversially discussed. Moreover, the pathomechanism of DD is not yet fully understood. Consequently, treatment is currently purely symptomatic, and various minimally invasive and open surgical methods have been described [18]. These include radiation therapy, percutaneous needle fasciotomy, the infiltration of enzymatic collagenase, and open surgical limited fasciotomy [19,20,21,22,23]. Open surgical limited fasciotomy is considered the gold standard in the current literature for the treatment of DD Europe [19,20,21]. It is particularly indicated for higher-grade contractures, the involvement of multiple fingers, severe movement restrictions in the proximal interphalangeal (PIP) and distal interphalangeal (DIP) joints, and recurrence treatment [14]. A disadvantage of the method is the longer postoperative recovery time due to wound healing and scar maturation. There is also the risk of complications such as wound healing disorders, skin necrosis, wound infections, persistent numbness, and injuries to arteries, nerves, or tendons [11].

As a non-invasive treatment for contractures in Dupuytren’s disease, enzymatic therapy with microbial collagenase offers a gentler approach. This therapy involves the intralesional injection of enzymes produced by the bacterium *Clostridium histolyticum*. Collagenase breaks down collagen types I and III. Since the cords in Dupuytren’s disease primarily consist of collagen type III, they are disintegrated by the collagenase, allowing the fingers to be straightened later under local anesthesia [24,25]. The product was marketed under the name Xiapex in the European Union since 2011, but its distribution has since been discontinued there. However, it continues to be marketed internationally under the name Xiaflex [24,26,27]. Due to the long recovery time associated with surgical limited fasciotomy and the high recurrence rate after minimally invasive needle fasciotomy, collagenase therapy appears to be a well-tolerated alternative. However, the data on this evolving therapy are still scarce, especially with regard to long-term results. We therefore set out to examine the long-term success of collagenase therapy in DD in a two-part study. In this first part, improvements in finger contracture and the rate of recurrence were analyzed. Additionally, the correlation of certain possible risk factors for Dupuytren’s disease concerning the long-term outcomes after collagenase therapy was examined.

## 2. Materials and Methods

For this monocentric retrospective study, patients were recruited who had undergone collagenase treatment for DD. All patients confirmed their voluntary participation in the study in writing and were over 18 years old. Approval for the study was obtained from the local ethics committee (Project Number: 18-265). The results of the early postoperative follow-up examinations were already published in 2017 by Pototschnig et al. [28].

### 2.1. Patient Cohort

Patients who were treated with collagenase between May 2011 and July 2014 were included in the study. The inclusion criteria were a Dupuytren’s contracture requiring therapy with a palpable nodule or cord. Patients who had previously undergone an alternative treatment for DD on the hand were excluded. Deceased or seriously ill patients, as well as patients who could not be contacted or declined further participation in the study, were also excluded from follow-up. Patients suffering a recurrence of Dupuytren’s disease during the follow-up period, which typically required limited fasciectomy, were followed up, but their clinical outcomes were not included in the evaluation of postoperative results after collagenase therapy.

### 2.2. Administration of Microbial Collagenase (Xiapex)

Xiapex is a combination of microbial collagenases class I and II (AUX-I, AUX-II), derived from Clostridium histolyticum [24]. According to the manufacturer, 0.9 milligrams (mg) of Xiapex powder and solvent were used to prepare an injection solution. Following the manufacturer’s recommendations, an injection volume of 0.25 milliliters (mL) was infiltrated into cords involving the metacarpophalangeal (MCP) joint, while 0.20 mL was used for those involving the PIP joint. The injection was administered intralesionally. The injection site was always chosen where the cord was furthest from the flexor tendon and not tightly adhered to the skin.

On a subsequent day, the cord was ruptured through the passive extension of the finger under local anesthesia with Mepivacain. For this, the patient’s wrist was held in a flexed position, while the treating physician applied moderate pressure on the cord by extending the finger for 10–20 s. The treatment was performed by an experienced hand surgeon trained in this procedure. Postoperatively, a palmar splint was applied to keep the fingers in an extended position until suture removal. Physiotherapy was prescribed for 2–4 weeks as well as a night positioning splint for 4 months postoperatively

### 2.3. Clinical Assessment of Dupuytren’s Disease

The extent of the contracture caused by Dupuytren’s disease at the affected joints was measured in degrees, as the total contracture of all joints in a finger, according to the classification developed by Tubiana and colleagues [11,12,13,14] (Table 1). A recurrence after therapy was defined according to the consensus statement by Felici et al. [29].

### 2.4. Statistical Analysis

The statistical analysis of the results was conducted using SPSS (ver. 27; International Business Machines Corporation, Armonk, New York, NY, USA). A significance level of *p* (*p*) ≤ 0.05 was chosen for all tests. To determine the effect size of a significant result, either effect seize Cohen’s d (d), effect seize Cohen’s f (f), effect seize r (r), or effect seize Eta-squared (η^2^) was calculated depending on the data. Table 2 provides the correct interpretation of the values.

For the evaluation of contracture improvements, depending on the number of measurement time points and the normality/non-normality of the data, Friedman’s two-way analysis of variance by ranks for related samples or the Wilcoxon test was applied. Differences in post-interventional contracture ratios between singly and multiply affected joints were determined using the Mann–Whitney U test.

To determine the influence of constitutional and lifestyle factors on long-term therapy outcomes, the corresponding correlation coefficients (Eta and Pearson coefficients) were calculated depending on the data, along with simple linear or multiple regression analyses and scatter plots. For this purpose, the respective percentage difference between the initial and final total contracture of each finger ray was calculated and used as a constant variable for the regression analyses. The individual risk factors were always included as independent variables.

## 3. Results

**Figure 1:**
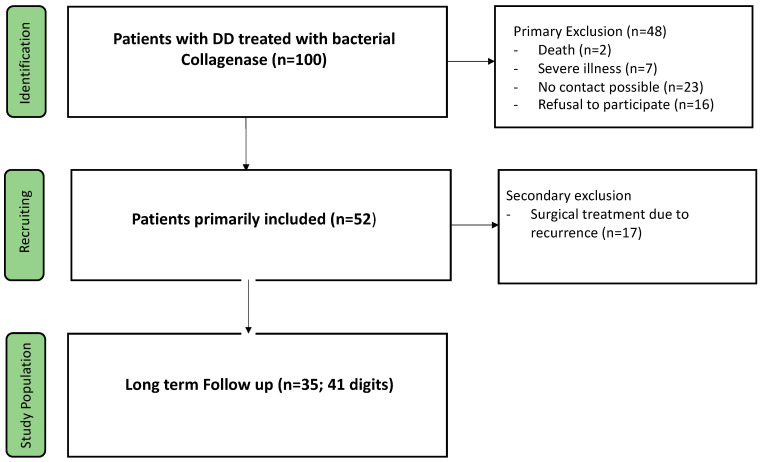
Study flow chart.

Out of the initial 100 patients, treated with collagenase, 52 could be included in the study. Out of these, 17 underwent subsequent surgical treatment for DD. These patients were counted as recurrence and were therefore only included in the assessment of potential risk factors. Thus, 35 patients, with a total of 41 digits, were included in the long-term clinical assessment after collagenase therapy (Figure 1). The mean time for follow-up was 68.4 (±7.2) months (5.7 (±0.6) years). The epidemiological characteristics of the patient collective are summarized in Table 3. We recorded no long-term adverse effects, particularly no neurapraxia/nerve injuries, arterial injuries, or flexor tendon ruptures, following collagenase treatment.

### Long-Term Results of Collagenase Therapy

The extent of the contracture caused by Dupuytren’s disease at the affected joints was measured at three assessment points (preoperatively, 6 weeks postoperatively, and 5.7 years after surgery (±0.6 SD)). Changes in each joint were documented in degrees, as well as the total contracture of all joints in a finger and the corresponding stage according to Tubiana. The results of the 6-week postoperative follow-up were derived from the previously published data of this patient cohort [28].

**Figure 2:**
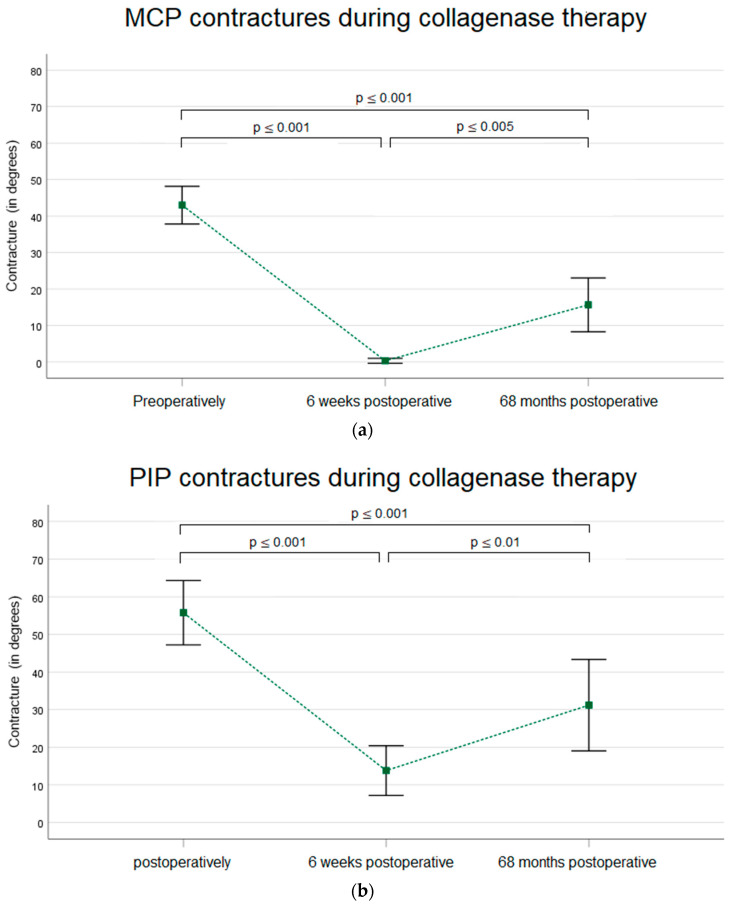
(**a**) MCP contracture of patients undergoing collagenase therapy over time at 3 measurement time points (*n* = 30). Data are expressed as means, and standard deviation bars are shown. Different superscripts indicate significant differences among groups. (**b**) PIP contracture of patients undergoing collagenase therapy over time at 3 measurement time points (*n* = 25). Data are expressed as means, and standard deviation bars are shown. (Data from the 6-week follow-up have already been published [28]).

In total, 30 MCP joints were re-evaluated at all three time points. These included both isolated MCP contractures and combined contractures. On average, the flexion contracture was 43° (±14°) preoperatively, 0° (±2°) at 6 weeks postoperatively, and 16° (±20°) after 5.7 years. With *p* < 0.001 (r = 0.17), the improvement in flexion contracture was also significant in the long-term follow-up (Figure 2a). A total of 25 PIP joints were re-evaluated at all three time points. On average, the flexion contracture was 56° (±21°) preoperatively, 14° (±16°) at 6 weeks postoperatively, and 31° (±29°) after 5.7 years. With *p* < 0.004 (r = 0.18), the long-term improvement in PIP flexion contractures was significant. (Figure 2b).

**Figure 3:**
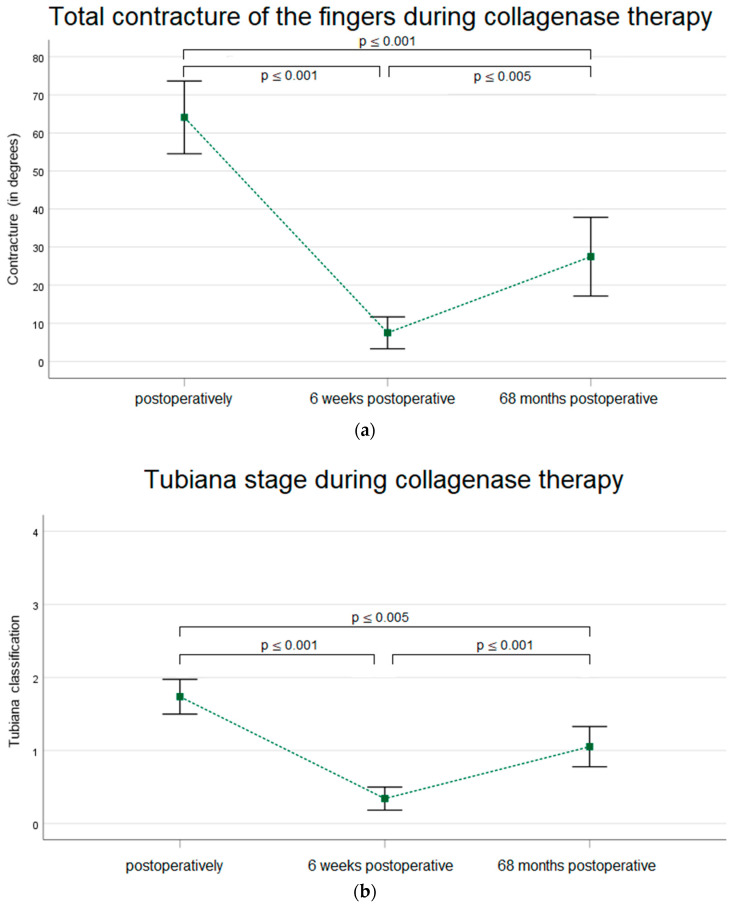
Developments of Dupuytren’s contracture according to (**a**) degrees of total contracture of the finger and (**b**) Tubiana stages (*n* = 38). Data are expressed as means, and standard deviation bars are shown. (Data from the 6-week follow-up have already been published [28]).

Considering the total flexion contracture per finger, an initial preoperative contracture of 64° (±29°) was observed. Six weeks postoperatively, it was 8° (±13°), and at the time of follow-up, it was 28° (±31°). The comparison of finger contracture preoperatively and at long-term follow-up showed a significant improvement in finger extension (*p* ≤ 0.001) (r = 0.168). According to the Tubiana classification, the stage before collagenase treatment averaged 1.7, six weeks postoperatively it was 0.3, and 5.7 years after treatment it was 1 (0–45° flexion contracture) (Figure 3).

**Figure 4:**
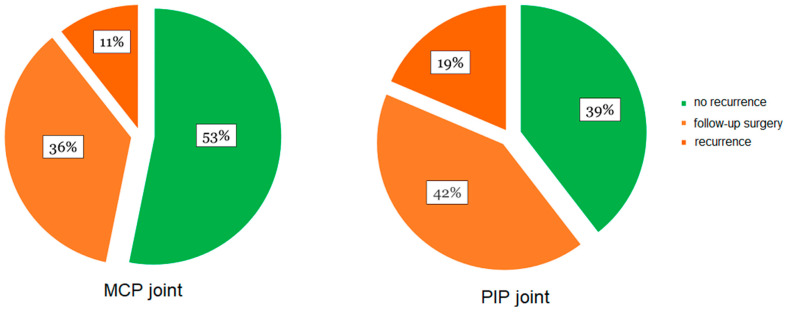
Recurrence rate in MCP and PIP joints 68 months after collagenase therapy. Recurrence rate shown in percent for the categories ‘no recurrence’, ‘follow-up surgery’, and ‘recurrence’ in *n* = 47 included MCP and *n* = 43 included PIP joints.

A worsening of Dupuytren’s flexion contracture by more than 20° compared to the result six weeks postoperatively was defined as a recurrence [29]. This was the case for 11% (5 joints) of MCP joints after 5.7 years. When including patients who had undergone surgical revision after collagenase treatment by the time of long-term follow-up examination, the recurrence rate increased to 47% (22 joints) (Figure 4). Likewise, PIP joints showed a recurrence of 19% (8 joints) and a total recurrence rate of 61% (34 joints) when including patients who had undergone surgical revision.

Impact of potential risk factors on the long-term evolution of Dupuytren’s contracture after collagenase therapy

As part of the follow-up examination, the presence of constitutional predispositions such as familial disposition, male gender, diabetes mellitus, hypercholesterolemia, and light eye color as well as lifestyle-dependent predispositions such as nicotine and alcohol abuse, and the amount of manual work were assessed (Table 4).

Association analyses were conducted between these potential risk factors and the difference in total contracture (preoperative vs. 5.7-year follow-up). We observed significant associations between a positive familial predisposition (*p* = 0.002; η = 0.799) and diabetes mellitus (*p* = 0.039; η = 0.361) (Table 4a). Consequently, regression analysis for these two risk factors also suggests that long-term outcomes are worse in these patients (*p* = 0.002, f = 1.33 and *p* = 0.039, f = 0.34, respectively). For nicotine consumption, correlation analysis showed a significant association with a worse long-term outcome of collagenase therapy (*p* = 0.017; r = 0.371) (Table 4b). Association and/or correlation analyses for male gender, hypercholesterolemia, blue or green eyes, alcohol consumption, and manual activity showed no negative impact of these risk factors on long-term outcomes.

## 4. Discussion

The existing data on the long-term effect of collagenase therapy are limited. Previous studies predominantly report on shorter observation periods of up to 32 months [24,31,32,33,34,35,36,37,38]. As the likelihood of a relapse or a recurrence increases over time, these studies predominately report a better improvement in contractures of MCP and PIP joints after treatment with collagenase, ranging between 40.5° and 6°. In our study, we report an improvement in flexion contracture of 27° (±20°) in MCP joints (*p* < 0.001 (r = 0.17) and of 25° (±22) in PIP joints (*p* < 0.004 (r = 0.18) 68.4 (±7.2) months after treatment (Figure 2a,b). This results in an improved overall flexion contracture of 36° (±29°) (*p* < 0.001) (r = 0.168) and of 0.7 (±0.6) points according to the Tubiana classification (*p* < 0.001, r = 0.443) of the affected digit (Figure 3a,b).

In an observation period comparable to our study, Werlinrud et al. followed up on 53 MCP joints after 5 years and 18 PIP joints after 4 years, achieving a percentage improvement in contracture of 88% and 77%, respectively [36]. In our study, we observed a relative improvement of 62.8% and 44.6% in MCP and PIP joints, respectively. The inclusion criteria in the study were singular strands with an isolated joint contracture and primary contractures without prior therapy [36]. In our collective, we also included combined joint contractures as well as Y-strands, representing a more realistic representative patient cohort. Watt et al. examined 8 patients after 8 years [38]. The authors describe similar long-term results for MCP joints and worse results for PIP joints. Thus, 2 isolated contracted PIP joints showed an increase in contracture from 45° to 60°. We observed an increased flexion contracture after 68 months in 5 PIP joints and 2 MCP joints. With low meaningful case numbers, it can only be speculated at this point as to whether there may also be a deterioration in the contracture in the treatment of PIP joints in the long term.

Nevertheless, in the increased recurrence rate of 61%, we observed that PIP joints imply an inferior outcome when compared to MCP joints (17%). Similar results were reported by a large multicenter study including 644 patients and 1081 joints to assess the recurrence rate of collagenase-treated patients (the CORDLESS study) [37]. Here, Peimer et al. showed a recurrence of 39% of MCP joints and 66% of PIP joints five years after collagenase therapy [37]. Using the same definition of recurrence, the follow-up study AUX-CC-860 after 5 years found a recurrence rate of 41% for MCP and 66.9% for PIP joints [24]. This phenomenon was also observed by studies with a shorter follow-up [24,25,39,40]. Therefore, it was concluded that the best results with collagenase therapy could be achieved in isolated MCP contractures [39,40]. Our results can confirm these findings.

Moreover, when reviewing the literature, we found only two studies with an observation period of five years or longer, the CORDLESS study and the above-mentioned study by Watt et al., who reported on the results of eight patients after 96 months (8 years) [37,38]. Our results therefore offer valuable insights into the long-term success of collagenase therapy for DD.

Interestingly, we identified significant risk factors that affect the long-term outcome of DD after collagenase therapy. Thus, in our study, we found that collagenase therapy was significantly less successful in patients with a familial predisposition, diabetes mellitus, and/or nicotine consumption (Table 4). These findings differ from the conclusions made by other authors. Clesham et al. and Raven et al. found no correlation between poor treatment outcomes and diabetes mellitus and/or family predisposition 1–3 months after injection nor with other risk factors such as epilepsy, gender, alcohol consumption, or family predisposition [41,42]. Importantly, a systematic review by Geoghegan et al. also found no negative impact of Dupuytren’s diathesis, nicotine abuse, and diabetes mellitus [43]. Simón-Pérez and colleagues, on the other hand, found a higher recurrence rate in a cohort of 71 patients after 4 years if the patient was younger than 60 years at the time of treatment [44]. The longer observational period in the study by Simón-Pérez as well as in the current study is a likely reason for this observed discrepancy. Indeed, the findings on the recurrence rate after collagenase therapy suggest that a final assessment of the success rate and most likely, the risk factors can only be performed in a long-term follow-up.

The small patient cohort must be seen as a major flaw in this study. Our study also suffered a considerable loss in follow-up. Since our clinic is a supra-regional hand surgery provider, the significant distance from patients’ residences could have prevented them from attending follow-up examinations. Additionally, there were problems contacting some patients due to changes in phone numbers or addresses during the study period, which were not communicated to the study leader. In particular, our findings on potential risk factors should be assessed in subsequent studies, possibly with a larger patient cohort. Moreover, the monocentric design of the study only offers insight into a relatively homogenous study population and may be a reason for the discrepancy of our findings with the results of the other main study with a similar long follow-up period (i.e., the CORDLESS study—multicentric) [37]. Nevertheless, to our knowledge, our study reports on one of the longest follow-up periods after collagenase therapy that can be found in the current literature. Moreover, our results offer an interesting insight into the possibility of identifying patients with a good prognosis for collagenase therapy, namely, predominantly, MCP contractures without the identified risk factors (familial predisposition, diabetes mellitus, and nicotine consumption). During the limited period when Xiapex was available for the treatment of Dupuytren’s disease in the EU, not all hand surgeons were trained or willing to use collagenase injection. Our hospital, as a supra-regional referral center for hand surgery, was one of the first to offer this therapy. Therefore, the conduct of the study by specially trained hand surgeons, as compared to the use of collagenase in general hand surgery practices, might pose a potential bias. Since our study specifically aimed at providing long-term results on the outcome of collagenase treatment for Dupuytren’s disease, patients suffering from recurrences were excluded from the evaluation of postoperative results. These patients are explicitly listed in Figure 4. As they were generally treated with limited fasciectomy, they must therefore be considered therapy failures of collagenase treatment.

An interesting subsequent assessment would be the comparison of long-term results of collagenase therapy to the established gold standard, namely limited fasciotomy. In a subsequent article, we will therefore compare the current patient cohort to patients who were treated with limited fasciotomy with a similar follow-up period with regard to recurrence rates and Patient-Reported Outcome Measures (PROMs).

## 5. Conclusions

Collagenase therapy for Dupuytren’s disease achieved significant long-term improvements in contracture in both MCP and PIP joints. In accordance with general risk factors for DD, patients with diabetes and those with nicotine abuse are at risk of worse long-term outcomes. Overall, it is a time-saving, low-risk, and straightforward technique for treating the disabling contracture component of this disease. A comparison to surgical treatment, particularly limited fasciotomy, with regard to postoperative outcomes, complications, and PROMs will be discussed in a subsequent article.

## Figures and Tables

**Table 1:** Classification of Tubiana for Stages of Dupuyren’s disease (adapted from Tubiana et al., 1968 [30]).

Tubiana Stage	Characteristics of Contracture
0/N	Aponeurotic lesion (nodule or band) no contracture
1	Overall contracture between 0 and 45 degrees
2	Overall contracture between 45 and 90 degrees
3	Overall contracture between 90 and 135 degrees
4	Overall contracture between 135 and 180 degrees

**Table 2:** Interpretation values of the respective effect sizes.

Effect Size	Effect Strength		
	Weak	Moderate	Strong
Cohen’s d (d)	<0.5	<0.5–0.8	>0.8
Cohen’s f (f)	0.1	0.25	0.4
Effect Size r (r)	<0.3	0.3–0.5	>0.5
Eta-squared (η^2^)	<0.06	0.06–0.14	>0.14

**Table 3:** Characteristics of the patient cohort.

Total	35
Male	28
Female	7
Age at the time of treatment (Mean ± SD)	68 ± 8.4
Affected digits	
Total	41
Middle finger	5
Ring finger	21
Little finger	15
Affected joints	
Total	55
MCP	30
PIP	25

**Table 4:** (**a**,**b**) Distribution of constitutional and lifestyle predisposition in patients treated by collagenase therapy (*n* = 35).

(**a**)					
**Risk Factor**	**Yes**	**No**	**No Data**		
Familial disposition	5	7	23		
Diabetes mellitus	4	25	6		
Smoking	10	25	0		
Male gender	28	7	0		
Hypercholesterolemia	13	16	6		
Blue or green eyes	16	13	6		
(**b**)					
**Risk Factor**	**None**	**Little**	**Moderate**	**Substantial**	**No Data**
Alcohol	6	10	12	7	0
Manual work	1	6	10	12	0

## Data Availability

The original contributions presented in the study are included in the article.
